# Candida Right-Sided Infective Endocarditis

**DOI:** 10.7759/cureus.97866

**Published:** 2025-11-26

**Authors:** Koshi Ota, Saneyuki Sumitomo, Kazuomi Sekine, Miwa Misawa, Tomio Suzuki

**Affiliations:** 1 Emergency and Critical Care Medicine, Osaka Medical and Pharmaceutical University, Takatsuki, JPN; 2 General Medicine, Osaka Medical and Pharmaceutical University, Takatsuki, JPN

**Keywords:** candida albicans, candida endocarditis, infective endocarditis, transthoracic echocardiography, tricuspid valve

## Abstract

Right-sided infective endocarditis (RSIE) is relatively uncommon, and *Candida* endocarditis is also rare, with high morbidity and mortality. We report a case of *Candida* tricuspid valve IE in a 78-year-old man with a native valve and no history of intravenous drug use. He presented with cough, chills, and malaise, and two blood cultures were positive for *Candida albicans*. Transthoracic echocardiography (TTE) revealed a mobile mass on the tricuspid valve. He was treated with antifungal and antibiotic agents and discharged on day 39, but later died of aspiration pneumonia. *Candida* endocarditis should be considered in patients with risk factors such as prior abdominal surgery and recent antibiotic exposure. TTE is an essential diagnostic tool for patients with persistent systemic symptoms.

## Introduction

*Candida* endocarditis is one of the most serious manifestations of candidiasis, with high morbidity and mortality. *Candida* species are the most common cause of fungal endocarditis [1]. In a large study of infective endocarditis (IE) in people who did not use injectable drugs, *Candida* species were reported in 1.5% of cases, a prevalence that remained stable over a six-year period [2]. The risk factors of *Candida* endocarditis include valvular disease or prosthetic heart valves, injectable drug use, indwelling central venous catheters, male sex, chronic immunosuppression (such as that resulting from cancer chemotherapy or solid organ transplantation), presence of an underlying right-sided cardiac anomaly, and prior bacterial endocarditis [1].

The clinical manifestations of *Candida* endocarditis are similar to those of bacterial endocarditis, but classic signs of IE, such as Osler nodes, Roth spots, and Janeway lesions, are uncommon [3]. Patients with right-sided IE (RSIE) may fulfill the 2023 Duke-International Society for Cardiovascular Infectious Disease criteria [4].

Due to the rarity of the condition, definitive evidence is weak, as prospective randomized trials are not feasible. Therefore, standard recommendations for *Candida* endocarditis, typically involving a combined approach of antifungal therapy and valve surgery, are based almost entirely on anecdotal reports, retrospective reviews, and expert opinion [1].

Here, we describe a case of *Candida* tricuspid valve endocarditis that was successfully treated with medical therapy alone.

## Case presentation

A 78-year-old man visited his primary care physician after experiencing fever and malaise for three days. A chest X-ray (CXR) at the primary care clinic showed infiltration, for which pneumonia was suspected, and he was prescribed an expectorant. His daughter subsequently became worried about his condition and brought him to our facility for further evaluation.

On arrival at the emergency room, his vital signs were as follows: temperature, 38.3°C; pulse, 100 beats per minute; respiratory rate, 18 breaths per minute; blood pressure, 79/41 mmHg; and oxygen saturation, 92% on room air. His Glasgow Coma Scale score was 15 (E4V5M6). He had multiple surgical histories and chronic obstructive lung disease with pneumonia.

The patient had a history of stage IV rectal cancer, which was managed four years ago with neoadjuvant chemoradiotherapy (tegafur-uracil (UFT) and calcium folinate hydrate (UZEL), and 40 Gy of radiation), followed by resection and stoma placement. An ileotransversostomy had subsequently been performed five months earlier to treat an ileus.

He denied sore throat, abdominal pain, or nausea, and could eat as usual. Testing of diarrheal stool samples for melena was positive. He had received pneumococcal vaccination four years previously. Physical examination revealed no abnormalities except for a coarse crackle in the right lung. No murmur or extra heart sound was present, and no peripheral signs such as Janeway lesions, Osler nodes, or Roth spots were observed.

**Figure 1:**
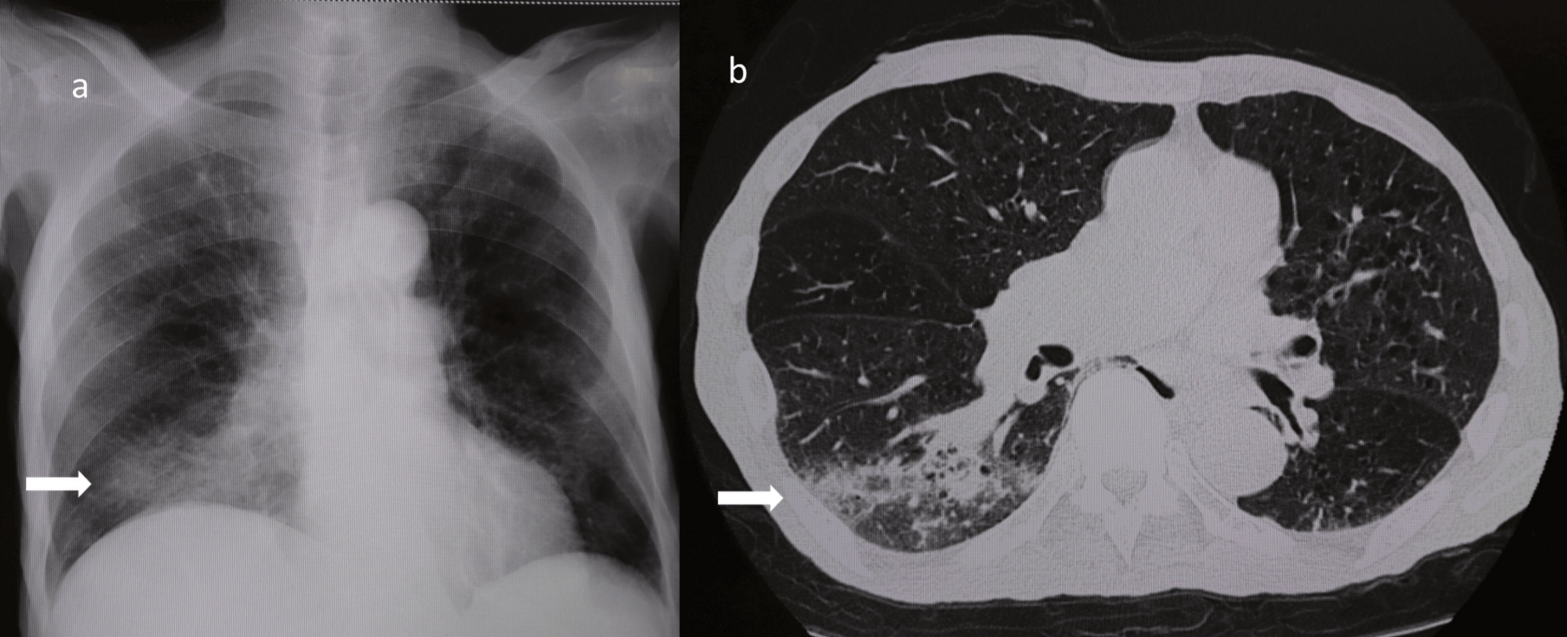
Chest radiographic findings a: Chest X-ray on admission showing right-sided pulmonary infiltration. b: Chest CT scan demonstrating right-sided pulmonary infiltration. CT: computed tomography

The pneumococcal urinary antigen test was positive. CXR and chest computed tomography (CT) showed right-sided infiltration (Figure 1). Antibiotic therapy with ampicillin-sulbactam (6 g/day) was initiated along with nasal oxygen therapy for right-sided pneumonia after sputum culture and two sets of blood cultures were obtained.

Initial laboratory examination revealed leukocytosis, elevated C-reactive protein (CRP), elevated D-dimer, and anemia, without thrombocytopenia or renal failure (Table 1). On hospital day 2, the serum 1,3-β-D-glucan level was elevated at 81.9 pg/mL (reference: ≤11 pg/mL). On hospital day 3, *Candida albicans* was detected in both sets of blood cultures (the isolate was susceptible to all agents on the panel).

**Table 1:** Initial laboratory findings on admission Laboratory tests on admission demonstrated leukocytosis, anemia, and elevated inflammatory markers. Reference ranges are provided in the right column. WBC: white blood cell, Hb: hemoglobin, Plt: platelet, Cr: creatinine, CRP: C-reactive protein

Parameter	Result	Reference range
WBC count	14,900/mm³	3,500-9,000/mm³
Neutrophils (%)	88.5%	40%-70%
Hb	7.4 g/dL	13-17 g/dL (male)
Plt count	243,000/µL	150,000-350,000/µL
Cr	0.71 mg/dL	0.6-1.2 mg/dL
CRP	10.8 mg/dL	<0.3 mg/dL
D-dimer	7.8 µg/mL	<0.5 µg/mL

**Figure 2:**
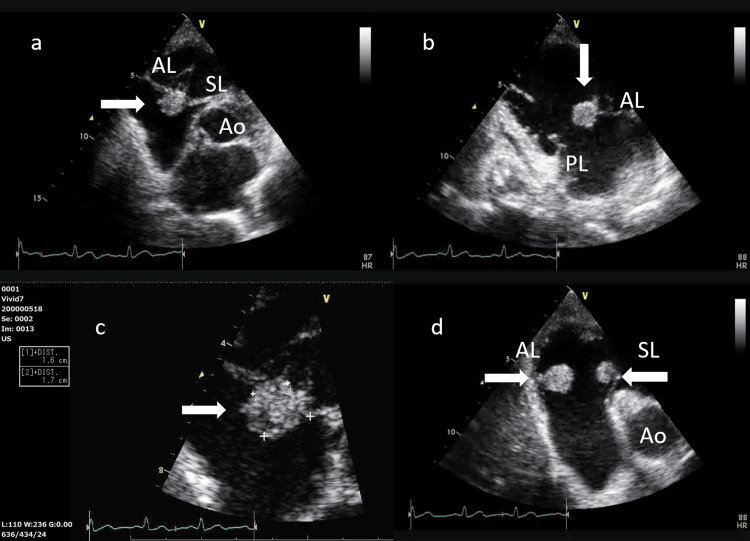
TTE (a to d) a: Arrow demonstrates the vegetation between the anterior tricuspid leaflet and the septal tricuspid leaflet during ventricular systole. b: Arrow shows that the vegetation involves only the anterior tricuspid leaflet. No vegetation is seen on the posterior tricuspid leaflet during ventricular diastole. c: The size of the vegetation was 16 mm × 17 mm during ventricular systole. d: Arrows showed separate vegetation on both the anterior tricuspid leaflet and the septal tricuspid leaflet during ventricular diastole. TTE: transthoracic echocardiography, Ao: aorta, AL: anterior tricuspid leaflet, PL: posterior tricuspid leaflet, SL: septal tricuspid leaflet

**Figure 3:**
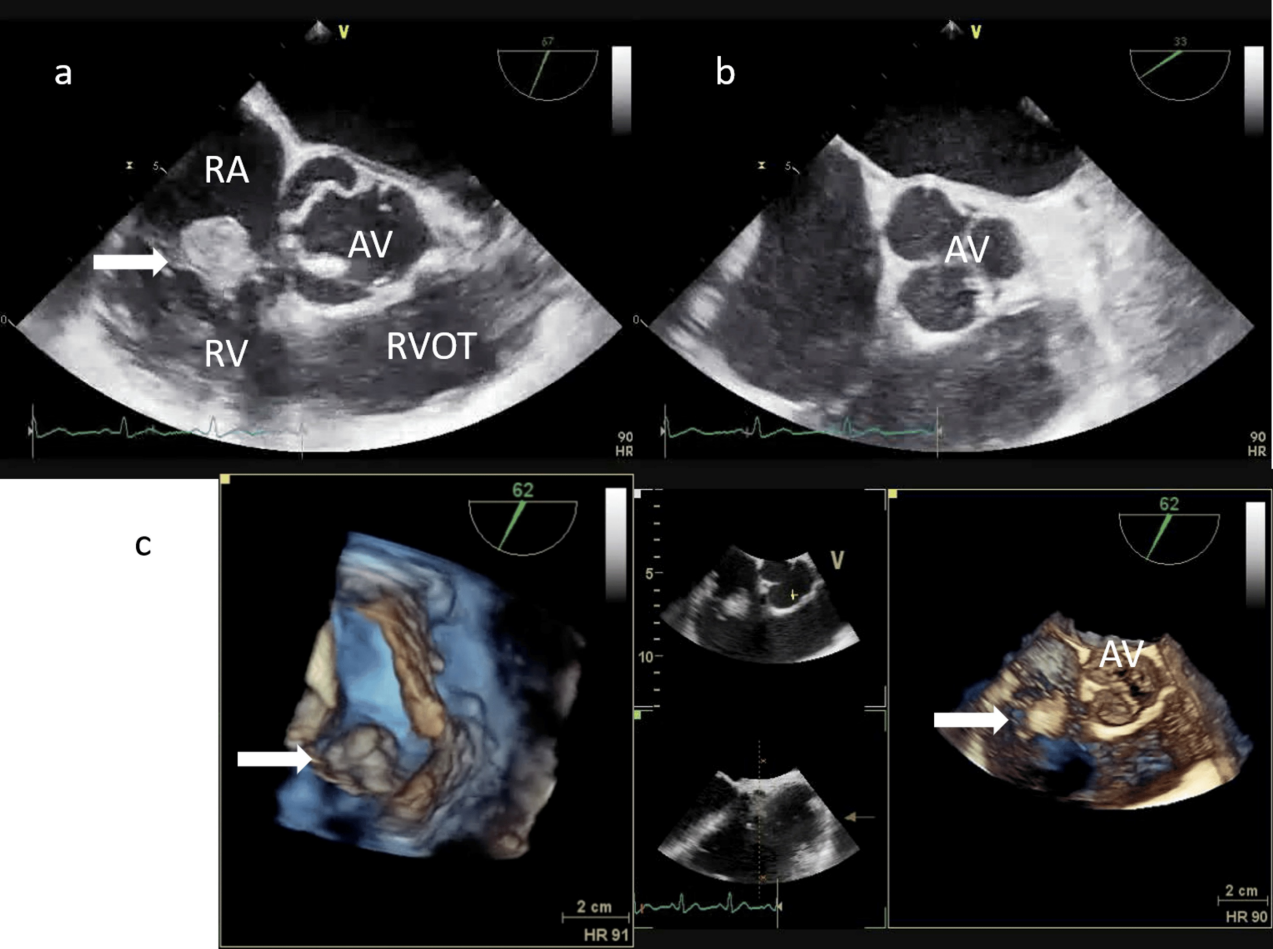
TEE (a to c) a: Arrow demonstrates the vegetation between the anterior tricuspid leaflet and the septal tricuspid leaflet during ventricular systole. b. Vegetation is not seen on the aortic valve. c: Three-dimensional TEE shows the vegetation between the anterior tricuspid leaflet and the septal tricuspid leaflet during ventricular systole. TEE: transesophageal echocardiography, AV: aortic valve, AL: anterior tricuspid leaflet, PL: posterior tricuspid leaflet, SL: septal tricuspid leaflet

Transthoracic echocardiography (TTE) revealed a highly mobile, hyperechogenic mass on the tricuspid valve (Figure 2). Antifungal therapy with amphotericin B (AmB) 250 mg/day was initiated the same day. Transesophageal echocardiography (TEE) performed on hospital day 5 confirmed valvular involvement and the absence of congenital heart disease (Figure 3).

Contrast-enhanced CT (CECT) did not show any emboli. Endophthalmitis was ruled out by an ophthalmologist. A cardiologist and cardiothoracic surgeon decided not to perform valve surgery because TEE did not show findings suggestive of heart failure and CECT did not reveal septic emboli.

**Figure 4:**
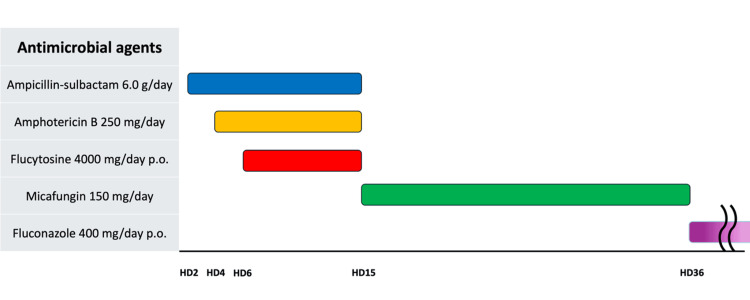
Antimicrobial agents Initial treatment involved ampicillin-sulbactam and AmB for two days. Flucytosine (4,000 mg/day) was added for nine days but was discontinued due to hypokalemia. Both ampicillin-sulbactam and AmB were subsequently stopped due to suspected drug eruption. The patient was transitioned to micafungin, which was continued until three days prior to discharge, followed by fluconazole therapy maintained throughout the subsequent follow-up period. HD: hospital day, AmB: amphotericin B

Ampicillin-sulbactam and AmB were administered for two days, after which flucytosine 4,000 mg/day was added for a further nine days. Flucytosine was discontinued because of hypokalemia, and ampicillin-sulbactam and AmB were also stopped because the patient developed eczema, which was suspected to be a drug eruption. Micafungin was initiated and continued until three days prior to discharge (Figure 4).

During this period, 1,3 β-D-glucan levels remained elevated at around 90 pg/mL, while CRP gradually decreased (Table 2). Three follow-up blood cultures performed during hospitalization were negative for all microorganisms, including *Candida albicans*. Fluconazole therapy was initiated three days before discharge and continued throughout the follow-up period (Figure 4).

The patient was discharged on hospital day 39 without complications. One month later, he was re-admitted for pneumonia caused by *Streptococcus pneumoniae*, which resolved with antibiotic therapy, and he was discharged uneventfully on hospital day 7. Multiple follow-up TTEs showed that the vegetation size remained stable and eventually underwent fibrosis.

**Table 2:** Time course of key laboratory markers WBC count, neutrophil percentage (%), Hb, CRP, and 1,3-β-D-glucan are presented. Despite the patient’s positive response reflected by a decrease in CRP and WBC, 1,3-β-D-glucan remained pathologically high, showing a higher value on the day of discharge (HD39) compared to admission. HD: hospital day, WBC: white blood cell, Hb: hemoglobin, CRP: C-reactive protein

Parameter	HD2	HD11	HD18	HD25	HD32	HD39 (discharge)	HD69 (re-admission)	Reference range
WBC count	12,580/mm³	5,150/mm³	4,400/mm³	5,240/mm³	6,280/mm³	5,180/mm³	10,040/mm³	3,500-9,000/mm³
Neutrophils (%)	89.10%	73.90%	65.20%	67%	73.50%	65%	91.40%	40%-70%
Hb	7.6 g/dL	6.8 g/dL	7 g/dL	8.7 g/dL	9.7 g/dL	9.8 g/dL	10.3 g/dL	13-17 g/dL (male)
CRP	10.93 mg/dL	7.05 mg/dL	3.15 mg/dL	0.53 mg/dL	2.35 mg/dL	1.98 mg/dL	7.27 mg/dL	<0.3 mg/dL
1,3-β-D-glucan	81.26 pg/mL	69.48 pg/mL	96.20 pg/mL	69.37 pg/mL	91.63 pg/mL	91.63 pg/mL	65.34 pg/mL	<0.5 µg/mL

## Discussion

Right-sided IE (RSIE) accounts for 5%-10% of all cases of IE, and most cases involve the tricuspid valve [5]. RSIE occurs mostly in injectable drug users (IDUs). RSIE in the non-IDU population occurs in individuals with permanent pacemakers, implantable cardioverter-defibrillators, central venous catheters, or congenital heart diseases [6]. However, one study reported that the source of IE was not detected in about 20% of patients with RSIE [5]. A fungal etiology has been reported as predictive of a negative outcome [6].

The prevalence of fungal IE is less than 10% of all IE, but is increasing because of the widespread use of external devices [1,7]. *Candida* species are the most common cause of fungal endocarditis, accounting for 1%-2% of all IE cases [7]. Patients with malignancy, a history of abdominal surgery, and antibiotic exposure within 30 days prior to *Candida* endocarditis have been reported to be more strongly associated with native valve endocarditis than with prosthetic valve endocarditis [8]. In our case, the patient had multiple abdominal surgeries for malignancy and received intravenous antibiotic therapy with a second-generation cephalosporin (flomoxef sodium) for six days within 30 days before admission for the treatment of gastroenteritis with pneumonia. These conditions may have triggered his *Candida* endocarditis, even though he did not have a prosthetic valve, external devices, or a history of injectable drug use.

A scientific statement has recommended valve surgery along with the use of a fungicidal agent in most cases of fungal IE [1]. In this case, the cardiologist and cardiothoracic surgeon decided not to perform valve surgery because CECT did not show septic emboli, TEE did not reveal severe regurgitation due to vegetation, and he responded to a fungicidal agent. While the surgical team was prepared to consider intervention for indications such as progressive valvular heart failure, systemic left-sided embolization, or failure of infection control, they judged that the patient’s anemic and malnourished state made the surgical risks outweigh the immediate benefits. However, vegetation of the tricuspid valve and high 1,3 β-D-glucan levels persisted despite long-term suppressive fluconazole therapy.

A lipid formulation of AmB with or without flucytosine is recommended as initial treatment for patients who cannot undergo surgical resection of the affected native valve [6]. Unfortunately, AmB had to be discontinued due to a drug eruption, and a high dose of micafungin was administered instead. A high dose of an echinocandin, including micafungin, has been recommended as an alternative to AmB with or without flucytosine. Notably, levels of the inflammation marker CRP were lowest during his treatment with micafungin. Although fluconazole is considered unsuitable as monotherapy for the initial treatment of *Candida* endocarditis, it is recommended for long-term suppressive antifungal therapy [1,9]. The patient’s condition remained relatively controlled by fluconazole suppression therapy, although 1,3 β-D-glucan levels remained persistently elevated for most of the clinical course.

However, CRP levels rose markedly at the time of both re-admissions. Moreover, the 1,3 β-D-glucan levels were lowest on the day of intensive care unit (ICU) admission but remained elevated upon re-admission for pneumonia with *Streptococcus pneumoniae*. This finding suggests that monitoring 1,3 β-D-glucan may not be a reliable indicator for tracking the activity or resolution of *Candida* endocarditis (Table 2).

The diagnosis of *Candida* endocarditis is often delayed owing to the prolonged duration of symptoms before hospitalization, absence of classic signs of IE (such as Osler nodes, Roth spots, and Janeway lesions), and extracardiac manifestations [3]. The increasing use of TTE has likely resulted in earlier diagnosis [3]. Although TTE should be the first diagnostic test for patients with suspected IE, its sensitivity is modest, reaching at most 75% [1]. Therefore, TEE is particularly useful for diagnosing RSIE and excluding other differential diagnoses when TTE is inconclusive and clinical suspicion for IE remains high.

We were unable to completely exclude other differential diagnoses, such as thrombus, bacterial endocarditis, or cardiac tumors, because we did not obtain histological confirmation of the vegetation. However, TTE and TEE demonstrated a large, dense, hyperechogenic mass adhering to the valve, which was compatible with *Candida* endocarditis. Blood cultures were also positive for *Candida* albicans. Thus, we believe that the mass on the tricuspid valve was vegetation caused by *Candida albicans*.

## Conclusions

*Candida *right-sided endocarditis accounts for less than 1% of all IE. A history of abdominal surgery and antibiotic exposure within 30 days are independent risk factors for *Candida* endocarditis in people without IDUs. In selected cases, our findings suggest that antifungal agents alone may suffice for native valve *Candida* endocarditis, despite standard recommendations. However, 1,3 β-D-glucan has limited utility for guiding management in patients with conditions promoting fungal translocation, such as recent abdominal surgery or sepsis. TTE is an important initial diagnostic tool for IE and should be considered for patients with high clinical suspicion for IE.
